# Supplementary material for: Associations Between GFAP, Aβ42/40 Ratio, and Perivascular Spaces and Cognitive Domains in Vascular Cognitive Impairment
Source: Int J Mol Sci. 2025 Apr 9;26(8):3541. doi: 10.3390/ijms26083541 (PMC12027347; doi:10.3390/ijms26083541)

Supplementary Materials

PVS_Total against Amyloid 42/40 Ratio

Variable	Value	Std. Error	t value	p value
Age	0.001186399	0.03797566	0.03124103	0.97507732
Gender	1.317140829	0.55003900	2.39463169	0.016663707
Diastolic BP	-0.02825806	0.0230846	-1.18583422	0.23568778
PSQI	-0.090253469	0.07985436	-1.13022588	0.25838106
Fazekas Total	0.171054873	0.13400216	1.27650836	0.20177587
Genotype	1.549010062	3.7285126	1.21690613	0.22363993
Amyloid 42 40 Ratio	-28.797508257	0.44378468	-64.8907221	**<0.00000001**

PVS_Total against GFAP

Variable	Value	Std. Error	t value	p value
Age	-0.010051167	0.041746360	-0.2407675	0.80937532
Gender	1.038439255	0.573304551	1.8113222	0.07009097
Diastolic BP	-0.033683368	0.024043786	-1.4009178	0.16123865
PSQI	-0.041583857	0.078854405	-0.5273498	0.59795068
Fazekas Total	0.200340006	0.132101946	1.5165561	0.12937881
Genotype	4.942921239	3.720180945	1.328085	0.18401017
GFAP mean concentration	0.011052	0.005483	2.015633	**0.043838**

PVS_Highest against Amyloid 42/40 Ratio

Variable	Value	Std. Error	t value	p value
Age	0.002238573	0.03806705	0.05880606	0.95310658
Gender	1.387877608	0.57330905	2.42081928	0.01548557
Diastolic BP	-0.027988630	0.02557840	-1.09422930	0.27385442
PSQI	-0.095924025	0.08734657	-1.09820019	0.27211708
Fazekas Total	0.177558290	0.13900404	1.27736065	0.20147495
Genotype	4.201356679	3.92866704	1.06941022	0.28488486
Amyloid 42 40 Ratio	-33.603171702	0.41957920	-80.08779146	**<0.00000001**

PVS_Highest against GFAP

Variable	Value	Std. Error	t value	p value
Age	-0.004664865	0.041634525	-0.1120432	0.91078916
Gender	1.150948156	0.596140511	1.9306659	0.05352438
Diastolic BP	-0.034640305	0.025311872	-1.3685398	0.17114318
PSQI	-0.054383628	0.085954026	-0.6327060	0.52692565
Fazekas Total	0.215308633	0.136972225	1.5719145	0.11597037
Genotype	4.453950684	3.884590256	1.1465690	0.25155980
GFAP mean concentration	0.01055066	0.005645228	1.8689514	0.06162958

Distribution of Amyloid 42/40 Ratio, GFAP mean concentration and PVS_Total

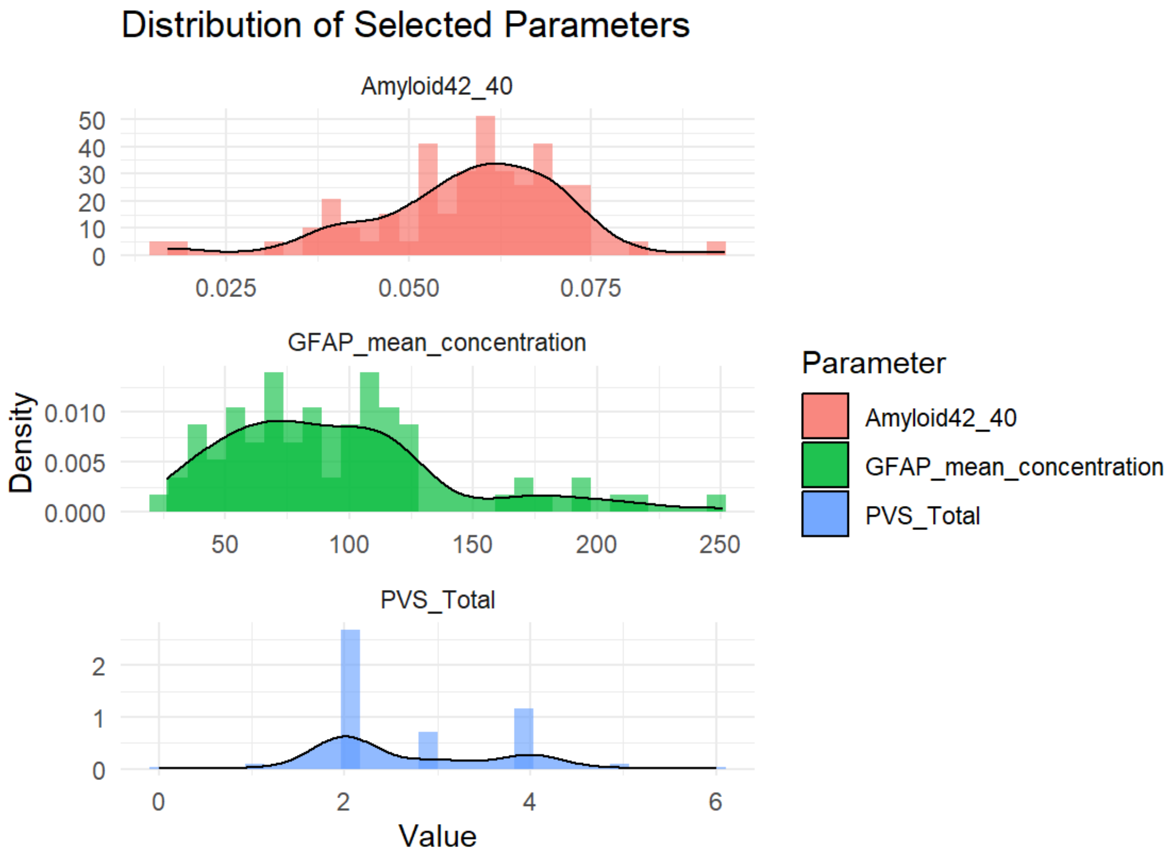

Supplement: Supplementary file 1 [file ijms-26-03541-s001.zip › ijms-3547168-supplementary.pdf]
